# Spontaneous Extensive Type A Aortic Dissection in an Older Female with No Risk Factors: A Rare Clinical Presentation

**DOI:** 10.1155/2023/4950510

**Published:** 2023-12-26

**Authors:** Maulik K. Lathiya, Susan M. Cullinan

**Affiliations:** Emergency Department, Mayo Clinic Health System, Eau Claire, WI, USA

## Abstract

**Background:**

Aortic dissection (AD) is a rare but serious medical emergency where the aorta's inner layer tears. Females are less likely to develop it than males, and AD cases with unusual symptoms can be hard to diagnose. Diagnosing AD can be further complicated as its symptoms and electrocardiogram (ECG) changes can mimic acute coronary syndrome, and it is challenging to distinguish it without risk factors. *Case Report*. This case report describes a 60-year-old female patient who presented with unusual symptoms, including pain in her chest, neck, left arm, and lower extremities. An electrocardiogram (ECG) revealed ST elevation in leads aVR and V1, as well as severe ST depression and T wave inversion in the inferior and lateral leads, which can mimic acute coronary syndrome. Despite initial treatment with nitroglycerin, the patient's pain worsened, and a CT angiography revealed type A aortic dissection extending from the aortic root to the right external iliac artery. Immediate surgery was recommended, which significantly improved the patient's condition.

**Conclusions:**

Be aware of aortic dissection and its symptoms, even if there are no risk factors or recognizable symptoms. Consider aortic dissection as a potential diagnosis if ECG changes are present. Ongoing education can help decrease mortality and increase awareness.

## 1. Background

Aortic dissection (AD) is a severe medical emergency caused by a tear in the inner layer of the aorta. Studies have shown that about 3 in 1,000 patients with chest, back, or abdominal pain are eventually diagnosed with acute aortic dissection. Although females tend to develop aortic dissection later in life, they are three times less likely than males to experience it [1]. However, about 25% of cases present with unusual symptoms that may be difficult to diagnose. This is further complicated by the fact that acute coronary syndrome can mimic AD due to similar symptoms and electrocardiogram (ECG) changes [2]. It can be particularly challenging to diagnose and distinguish AD when there are no risk factors present, such as hypertension, which is the most common risk factor. We present a case of a female patient with atypical ECG changes who was successfully treated for Stanford type A aortic dissection.

## 2. Case Presentation

A 60-year-old woman presented to the Emergency Department (ED) with complaints of neck pain, left arm pain, and lower extremity pain. The chest discomfort started at home but had subsided by the time Emergency Medical Services (EMS) arrived at the ED. Upon arrival, she was profusely sweating, pale, and in distress, with pain and weakness in her right leg as well as paresthesias. Despite her distressed and pale appearance, she was alert and oriented and able to provide a complete medical history. Her pulmonary, cardiac, and abdominal examinations were unremarkable, and her upper extremity pulses were strong. At first, she had decreased pulses in her right lower extremity, but this worsened over time, resulting in no pulses and a cold, mottled right lower extremity. The patient had no previous medical conditions, such as hypertension or hypercholesterolemia, and no family history of heart disease. She also had no personal or family history of connective tissue disease, hereditary disorders, bicuspid aortic valve, prior episodes of aortic aneurysm, or dissection. Her vital signs were stable in the ED, with a blood pressure of 106/84 mmHg, a pulse rate of 69 beats per minute, a respiratory rate of 21 beats per minute, a body temperature of 34.3 degrees Celsius, and oxygen saturation of 100%.

## 3. Investigations

**Figure 1:**
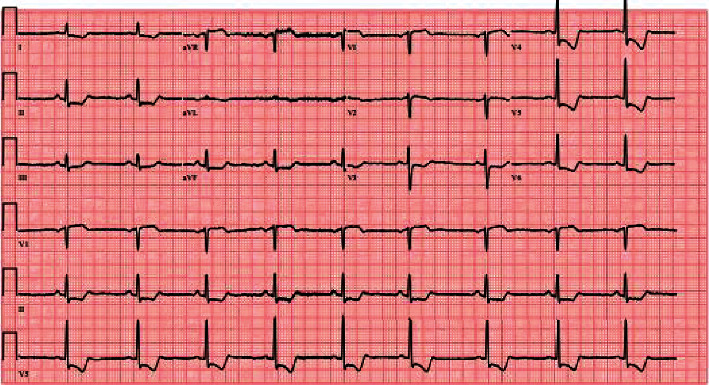
Initial ECG of ST-T wave abnormalities. Leads aVR and V1 show ST-segment elevation. Leads I, II, III, aVL, aVF, and V3-V6 show ST-segment depression and T wave inversion.

**Figure 2:**
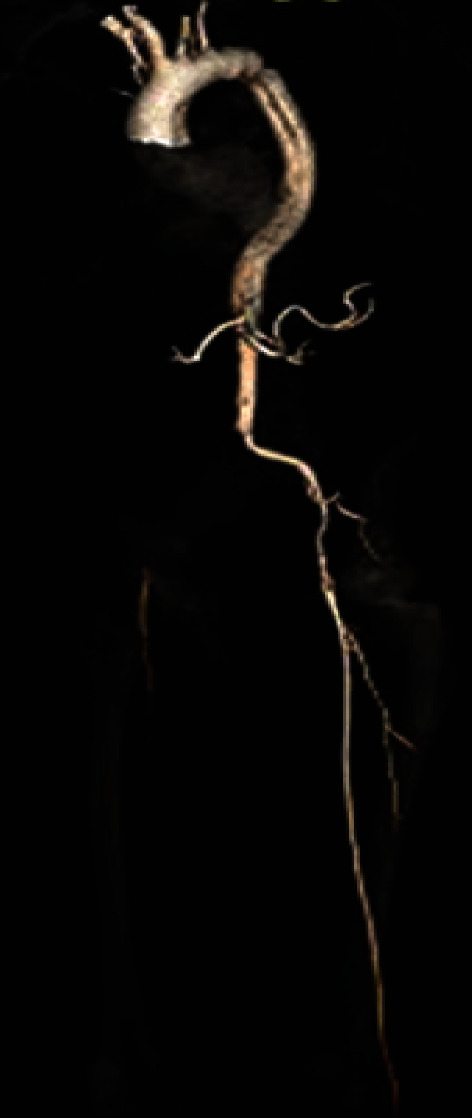
Anterior view of 3D computed tomography (3D CT) imaging of entire aortic artery with obvious dissection of ascending aorta, arch of aorta, right subclavian artery, and right common iliac artery.

**Figure 3:**
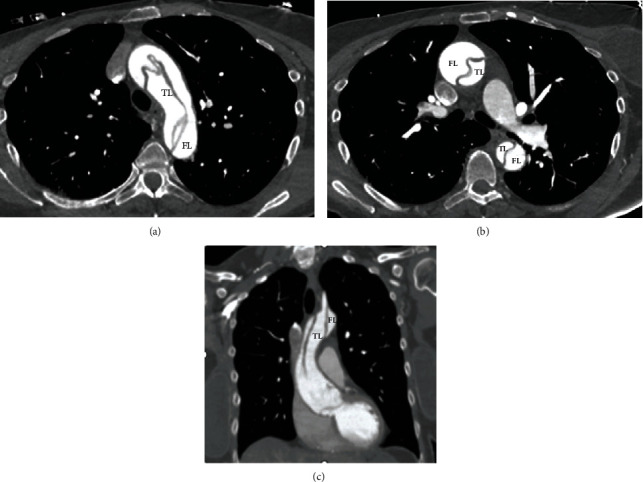
Computed tomography (CT) angiography showing aortic dissection in axial view (a, b) and dissection coronal view (c). FL: false lumen; TL: true lumen.

An ECG was conducted, and it revealed ST elevation in leads aVR and V1, severe ST depression, and T wave inversion in the inferior and lateral leads (Figure 1). Because the ECG results were suspicious for acute coronary syndrome (ACS), the patient was evaluated by a cardiologist in the Emergency Department. During this time, the patient's lower leg pain became more intense and gradually became mottled. As a result, a CT angiography was recommended and conducted, which revealed a type A aortic dissection extending from the aortic root to the right external iliac artery, without involving the coronary arteries. There was limited visualization of the left anterior descending artery with suspicion of involvement by the dissecting flap (Figures 2 and 3). The dissection involved the brachiocephalic artery, right common carotid artery, and right subclavian artery. It was discovered that the proximal right subclavian artery had significant stenosis. The dissection also extended into the origin of the left common carotid artery and left subclavian artery, and the flap of dissection extended to the bifurcation of the aorta into the right common iliac artery.

Additionally, the celiac artery, superior and inferior mesenteric arteries, and the right renal artery were also incorporated in the flap (Figure 2). Furthermore, the intraoperative echocardiography showed a normal trileaflet valve, but severe aortic valve regurgitation resulting from prolapse of the dissection flap.

## 4. Differential Diagnosis

Prior to imaging or surgical procedures were conducted, the possible causes of chest discomfort following an ECG included ST elevation myocardial infarction (STEMI), among other potential diagnoses.

## 5. Treatment

Based on the initial findings of the ECG, the ST elevation myocardial infarction (STEMI) protocol was initiated, and the patient received treatment with a nitroglycerin bolus and infusion. Although the patient's chest pain improved, her pain in the right leg worsened, along with worsening mottling and absence of pulses. After a CT scan revealed that the patient had a type A AD, the heparin infusion was immediately discontinued, and cardiovascular surgeons advised immediate surgery. Before the surgery, the patient's left ventricular function was approximately 20%, with significant anterior wall akinesis attributed to a dissected left main takeoff. Due to the anticipated difficulty of repair, the patient was transferred to a higher level of care. Throughout her course, the patient's systolic pressure remained within the range of 90-110 mmHg, and she did not require any blood pressure medications. After being transferred, the patient underwent an initial sternotomy, aortic valve resuspension, ascending aortic and hemiarch replacement, reimplantation of the innominate artery, and implantation of a stent graft to the descending thoracic aorta.

## 6. Outcome and Follow-Up

After undergoing ascending aortic and hemiarch replacement, there was an improvement in blood flow to the coronary ostia with trial residual aortic valve regurgitation. This made it unnecessary to perform coronary artery bypass grafting, and the patient's left ventricular function improved to 45%. Following the surgery, the patient's condition stabilized, and she was then transferred to the intensive care unit. The patient's right lower extremity showed improvement with reduced pain, palpable pulses, and full function. The patient was discharged one week after admission and will undergo chest CT angiogram at 6 and 18 months to monitor the progress of the aortic graft repair.

## 7. Discussion

The Centers for Disease Control and Prevention (CDC) conducts an annual “National Ambulatory Medical Care Survey,” which reveals that about 7 million patients with chest pain visit the Emergency Department (ED) every year in the United States, accounting for 5.5% of all ED patients [3]. There are numerous life-threatening and non-life-threatening causes of chest pain, which are complicated by differences in signs, symptoms, underlying pathology, and severity. Some important causes of chest pain in the ED are acute coronary syndrome, aortic dissection, pulmonary embolism, pneumothorax, pericarditis, gastroesophageal reflux disease, and esophageal rupture [4]. However, identifying the underlying cause of chest pain can be challenging, particularly in the case of aortic dissection, which is often misdiagnosed. Patients with atypical symptoms, a history of heart failure, concomitant aortic insufficiency, or prior cardiac surgery, especially older women, are more difficult to diagnose correctly [2]. Due to the nature of the pathology, misdiagnosis and delay in treatment can have a significant impact on patient outcomes. Low prevalence and a lack of treatment guidelines are among the existing diagnostic challenges for AD. The similarity to acute coronary syndrome, stroke, or pulmonary embolism is particularly concerning and can lead to the administration of inappropriate treatment. Despite advances in imaging modalities and biomarkers, acute aortic dissection is frequently misdiagnosed.

**Figure 4:**
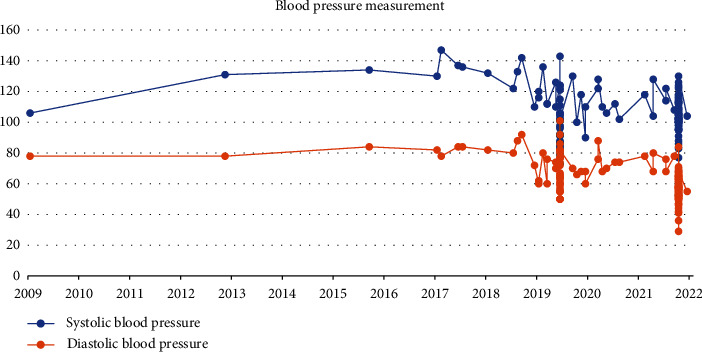
Blood pressure measurements on patient from 2009 to 2022 demonstrating no hypertension history.

In addition to these diagnostic pitfalls, when evaluating patients with chest pain, it is important to consider underlying comorbidities and risk factors that may lead to aortic dissection (AD). AD risk factors may include hypertension, atherosclerosis, dyslipidemia, smoking, connective tissue disorders, cardiac valve disease, a family history of aortic disease, sympathomimetic agents, trauma, and vascular inflammation, of which hypertension is the most important and common risk factor [5, 6]. According to Lovatt et al., hypertension (55.4%), a smoking history (30.5%), coronary artery disease (14.7%), and Marfan syndrome (3.6%) are the most prevalent underlying risk factors for AD [7]. Reference [7] examined over a million patients to determine a link between hypertension and aortic dissection and discovered that hypertension is the most prevalent cause of aortic dissection. In [8], patients who died within three years of surgery had higher systolic blood pressures, whereas patients on beta-blocker therapy required less reoperation when their blood pressure was controlled during late follow-up. Therefore, identifying risk factors is crucial in determining the underlying cause of aortic dissection and subsequent management. However, patients without significant risk factors can present a challenge for diagnosis, leading to a delay in treatment and potentially affecting the prognosis of both aortic dissection and acute coronary syndrome. For instance, our patient (Figure 4) did not have a history of hypertension, take antihypertensive medication, or have any other potential risk factors for AD.

In medical literature, it is emphasized that the electrocardiogram (ECG) is an important diagnostic tool in addition to a patient's medical history and physical examination. Ischemic ST-T abnormalities can result from various conditions, including acute ischemia due to AD or underlying hypertension, certain heart conditions such as pericardial effusion, cardiac tamponade-induced global ischemia, shock, or valvular abnormalities. Of which, acute coronary syndrome-related ST-T abnormalities are often misdiagnosed as AD, delaying treatment or mistreatment [9]. Kosuge et al. reported that 51% of patients with aortic dissection had ST-T abnormalities, with only 4% experiencing ST-segment elevation and 47% experiencing ST-segment depression and/or T wave inversion. Patients with ST-T wave changes had a higher incidence of aortic dissection-related complications such as pericardial effusion, shock, cardiac tamponade, in-hospital mortality, and emergency surgery for aortic dissection. In addition, ST elevation and T wave inversion are associated with increased in-hospital mortality and poorer outcomes in patients with aortic dissection, as well as misdiagnosis, which can lead to fatal complications from fibrinolytic therapy. Furthermore, patients with ST-segment elevation in the aVR lead are more likely to experience cardiac tamponade, shock, or involvement of the origin (ostial) portion of the coronary artery and have a higher in-hospital mortality rate [9]. In this instance, the patient's ECG demonstrated ST-segment depression and T wave inversion in all leads except aVR and V1, which demonstrated ST-segment elevation. Based on these findings, the patient was initially treated for acute coronary syndrome with heparin and nitroglycerin. However, following the diagnosis of aortic dissection by computed tomography angiography, these medications were immediately discontinued.

## 8. Conclusion

Aortic dissection can present with diverse symptoms that can imitate other common conditions. Therefore, it is crucial to maintain a high level of suspicion, even in the absence of risk factors or recognizable symptoms for AD. Moreover, ECG changes, such as diffuse ST depression and T wave abnormalities, may indicate AD as a cause of chest pain. Continual clinical education regarding these manifestations can decrease overall mortality and increase awareness of AD.

## Data Availability

All relevant data are included in the report and the associated images. Further inquiries can be directed to the corresponding author.
